# The microtubule-associated protein tau is phosphorylated by Syk

**DOI:** 10.1016/j.bbamcr.2007.11.005

**Published:** 2008-02

**Authors:** Thibaud Lebouvier, Timothy M.E. Scales, Diane P. Hanger, Robert L. Geahlen, Bernard Lardeux, C. Hugh Reynolds, Brian H. Anderton, Pascal Derkinderen

**Affiliations:** a Inserm, U643, Nantes, F-44000, France; b Department of Neurology, CHU de Nantes, F-44000, France; c Université de Nantes, Faculté de Médecine, Nantes, F-44000, France; d MRC Centre for Neurodegeneration Research, Department of Neuroscience, Box 037, King's College London, Institute of Psychiatry, London SE5 8AF, UK; e Department of Medicinal Chemistry and Molecular Pharmacology, Purdue University, West Lafayette, Indiana, USA; f Inserm, U913, Nantes, F-44000, France

**Keywords:** Tau, Tyrosine phosphorylation, Syk, Tauopathy

## Abstract

Aberrant phosphorylation of tau protein on serine and threonine residues has been shown to be critical in neurodegenerative disorders called tauopathies. An increasing amount of data suggest that tyrosine phosphorylation of tau might play an equally important role in pathology, with at least three putative tyrosine kinases of tau identified to date. It was recently shown that the tyrosine kinase Syk could efficiently phosphorylate α-synuclein, the aggregated protein found in Parkinson's disease and other synucleinopathies. We report herein that Syk is also a tau kinase, phosphorylating tau *in vitro* and in CHO cells when both proteins are expressed exogenously. In CHO cells, we have also demonstrated by co-immunoprecipitation that Syk binds to tau. Finally, by site-directed mutagenesis substituting the tyrosine residues of tau with phenylalanine, we established that tyrosine 18 was the primary residue in tau phosphorylated by Syk. The identification of Syk as a common tyrosine kinase of both tau and α-synuclein may be of potential significance in neurodegenerative disorders and also in neuronal physiology. These results bring another clue to the intriguing overlaps between tauopathies and synucleinopathies and provide new insights into the role of Syk in neuronal physiology.

Many neurodegenerative disorders are characterised by intracellular inclusions of highly insoluble proteins, and a classification based upon the main protein component of these aggregates is widely used. The term “synucleinopathies”, for instance, refers to a group of disorders including Parkinson's disease (PD) in which the synaptic protein α-synuclein (α-syn) forms neuronal or glial aggregates [1]. “Tauopathies” are another pathological entity in which the microtubule-associated protein tau self-assembles into filamentous inclusions called neurofibrillary tangles (NFT) [2]. The tauopathies include Alzheimer's disease (AD), progressive supranuclear palsy and corticobasal degeneration.

Human brain tau consists of a family of six isoforms, generated by the alternative splicing of a single mRNA transcript. Tau proteins contain either three (tau 3R) or four (tau 4R) tubulin-binding domains depending on the splicing of exon 10, and either one (tau 1N) two (tau 2N) or no (tau 0N) N-terminal inserts depending on the inclusion of exon 2, exons 2 and 3, or exclusion of both, respectively. Their apparent molecular weights range from 45 kDa for tau 0N3R isoform to 65 kDa for tau 2N4R. When aggregated in NFT, tau is abnormally hyperphosphorylated by a series of serine/threonine kinases, among which glycogen synthase kinase-3β, cyclin-dependent kinase 5 and casein kinase 1 appear to be the most relevant [3,4]. It has been hypothesized that this hyperphosphorylation contributes to neurodegeneration through the destabilisation of microtubules [5].

Recent data suggest that phosphorylation of tau also occurs on tyrosine residues and this could be of potential significance in pathological conditions [6,7]. Human tau protein possesses five tyrosine residues on positions 18, 29, 197, 310 and 394 (numbered according to the longest 2N4R tau isoform). Tau extracted from NFT of AD patients was found to be phosphorylated on tyrosine 18 (Y18) by immunocytochemistry using a phosphospecific antibody [6], and on tyrosine 394 (Y394) by mass spectrometry [8]. Tyrosine 197 (Y197) has been shown to be phosphorylated in tau aggregates from transgenic mice overexpressing mutant tau [9]. Recently, the dual specificity kinase tau-tubulin kinase 1 has been shown to phosphorylate tau *in vitro* on Y197 [10]. While Abl was recently described as a candidate tyrosine kinase for Y394 [8], Fyn is considered to be the kinase for Y18 [6].

The tyrosine kinase Syk is known for its critical role in signalling through immune receptors in leucocytes [11]. Syk tyrosine kinase expression is however not confined to hematopoietic cells, and has been reported in a variety of tissues [12]. Syk expression has been confirmed in the central nervous system (CNS) by immunoblot analysis of mouse brain homogenates, and its localization in mouse neuronal cytoplasm affirmed by immunohistochemistry [12]. While its function in the CNS remains unclear, Syk was recently found to be a kinase for α-syn [13]. The direct interaction of Syk and α-syn was proven by confocal microscopy and a dual-hybrid system approach [13]. The intriguing overlap in the pathophysiology of synucleinopathies and tauopathies prompted us to assess whether tau protein was also a substrate for Syk tyrosine kinase [14]. We herein report that Syk can phosphorylate Tau on Y18, and that the kinase and its substrate directly interact.

We first investigated whether Syk can directly phosphorylate tau *in vitro*. Recombinant human tau 2N4R was incubated with or without recombinant Syk or Abl [8]. Tau was phosphorylated *in vitro* by Syk and by its previously recognized tyrosine kinase Abl as judged on Western blots probed with P-Tyr-100 anti-phosphotyrosine antibody (Cell Signaling), whereas no phosphorylation was observed in experiments where tau was incubated without Syk (Fig. 1). In the presence of Syk, an additional band of approximately 70 kDa, migrating just above tau, was observed on the phosphotyrosine immunoblot. This band is likely to contain Syk since the kinase is known to autophosphorylate. Taken together, these results demonstrate that Syk can directly catalyze tau phosphorylation.

To determine whether Syk can phosphorylate tau in cells, co-transfection experiments were performed in CHO cells using Syk expression vectors together with untagged or V5-tagged tau. Cells were lysed and tau was immunoprecipitated with either anti-V5 antibody (Invitrogen) or with anti-tau antibody (Tau-5, BD Biosciences). Western analysis was then performed on immunoprecipitated tau using the anti-phosphotyrosine (P-Tyr-100, Cell Signaling), anti-tau (Tau-5) or anti-Syk (N-19, Santa Cruz) antibodies as previously described [8]. In preliminary experiments, a V5-tagged tau 2N4R construct and wild-type Syk were transiently coexpressed in CHO cells. We have previously shown that V5-tagged tau 2N4R migrates at approximately 70 kDa on SDS-PAGE [8]. Because of the close molecular weights of Syk (72 kDa) and the V5-tau construct, the two proteins co-migrated using conventional denaturing gel electrophoresis (data not shown). To better resolve the two proteins, we used two complementary approaches. In a first set of experiments, we expressed V5-tagged tau 2N4R along with a Syk-GFP construct that migrates at 100 kDa. The Syk-GFP construct was proven to remain functionally active in previous studies [15]. In a second set of experiments, we coexpressed wild-type untagged Syk along with the shortest tau isoform (0N3R), which migrates at 45 kDa. Tau protein was phosphorylated on tyrosine when co-transfected with Syk-GFP construct or with its known tyrosine kinase Abl (Fig. 2A). Additional positive controls included tau-transfected cells treated with the tyrosine-phosphatase inhibitor pervanadate (100 μM for 30 min). When Syk-GFP was co-transfected with tau, tau immunoprecipitates contained a ∼ 100-kDa tyrosine-phosphorylated protein, likely to contain Syk-GFP since this enzyme is tyrosine phosphorylated (Fig. 2A). This was confirmed by blotting with Syk antibody after membrane stripping (Fig. 2A). This co-immunoprecipitation of Syk with tau is indicative of Syk and its substrate being in the same protein complex. In order to exclude any effects of the V5 and GFP tags fused with tau and Syk, we wished to repeat this experiment using vectors expressing native proteins. The untagged and shortest isoform of tau (0N3R) was phosphorylated on tyrosine when co-transfected with native 72 kDa Syk (Fig. 2B), as compared with the transfection of tau alone. As in Fig. 2A, an additional 72-kDa band corresponding to phospho-Syk was visible on the phosphotyrosine blot when Syk was co-transfected with tau. The co-immunoprecipitation is therefore confirmed with native proteins. Taken together, these results demonstrate that Syk tyrosine kinase can both phosphorylate and co-immunoprecipitate with tau in cells, consistent with the direct phosphorylation of the substrate demonstrated *in vitro* (Fig. 1). The binding of Syk to both the shortest and longest isoform of tau suggests that it is independent of tau splicing.

Tau has been shown to be tyrosine phosphorylated on residue 18, 197 and 394 [6,8–10]. To map the tyrosine residue(s) phosphorylated by Syk in tau, Syk was co-transfected into CHO cells along with wild-type or mutant tau constructs in which individual tyrosines were replaced by phenylalanine (Y18F, Y197F and Y394F). These cDNA constructs have been described in detail previously [8]. The only such tyrosine mutation that resulted in a strong decrease in tau tyrosine phosphorylation was Y18F (Fig. 3). Tyrosine phosphorylation of the Y197F and Y394F constructs were not significantly different from the wild-type control (Fig. 3). This clearly demonstrates that Y18 is the major tyrosine phosphorylation site for Syk. In addition, the binding of Syk was independent of the tyrosine phosphorylation state of tau (Fig. 3), suggesting that the SH2 domains of Syk are not involved in its binding to tau.

As Fyn and Syk phosphorylate the same tyrosine residue on tau, we decided to perform a time course of phosphorylation of tau by the two kinases under conditions designed to favour the measurement of stoichiometry. After 30 min, Syk had incorporated an average of 0.23 mol of phosphate/mol of tau and Fyn had incorporated an average of 0.25 mol of phosphate/mol of tau. These results show that Syk and Fyn phosphorylate tau with the same efficacy (Fig. 4).

Our demonstration of binding of tau to Syk is consistent with tau being a specific substrate for Syk and for their involvement in a cell-signaling pathway in neurons. Interestingly, Syk plays an important role in signalling events of neurite induction and outgrowth in neuronal cell lines [16]. It is thus tempting to speculate that the phosphorylation of tau by Syk could be involved in neurite outgrowth. Our study also reinforces the role of Syk in neurodegenerative disorders. Remarkably, Y18 has been shown to be phosphorylated in NFT, suggesting that, like Fyn, Syk could be critical in the pathophysiology of AD [6,7,17]. Although early studies suggested a clear distinction between ‘tauopathies’ and ‘synucleinopathies’, more recent studies demonstrate that there is often an overlap in the pathological findings in these disorders [18–20]. Syk binds to and phosphorylates both tau and synuclein, suggesting that this kinase could be a link between synucleinopathies and tauopathies. In conclusion, our findings significantly contribute to the understanding of the signaling pathways involving tyrosine phosphorylation of tau in both physiological and pathological conditions. Further elucidation of the functional relationship between tau and the Syk in neurons could provide critical insights into the role of tau in cell signaling as well as the role of tau in neurodegenerative processes.

## Figures and Tables

**Fig. 1:**
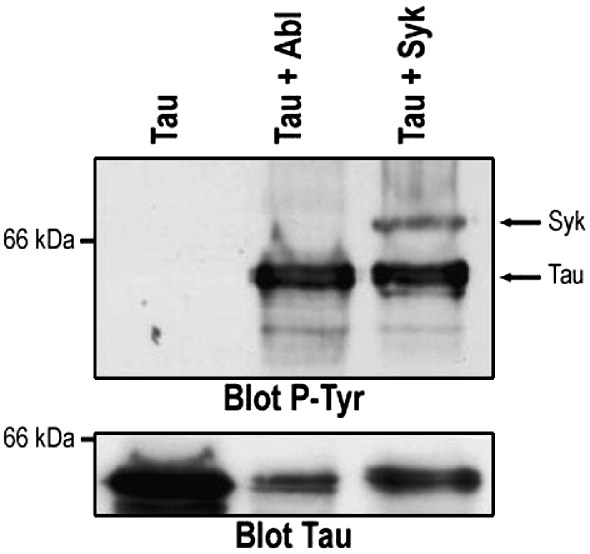
Phosphorylation of tau by Syk *in vitro*. Recombinant tau 2N4R was expressed in *E. coli* BL21 (DE3) and purified as described previously [21]. Recombinant human tau (2 μg) was incubated with or without 0.4 μg of Syk or Abl (Invitrogen) in 30 μl of kinase buffer (50 mM Hepes pH 7.4, 5 mM MnCl_2_, 5 mM MgCl_2_, 0.01% (v/v) Triton X-100, 12 mM beta-glycerophosphate and 1 mM dithiothreithiol) in the presence of 0.2 M ATP for 30 min at 30 °C. 30 μl of SDS-PAGE sample buffer was added to stop the reaction. Control tau was put through the same procedure but without kinase. The phosphorylation reactions were analyzed by Western blotting using 8% gels and P-Tyr-100 (Blot P-Tyr) and tau-5 (Blot Tau). The data presented are representative of three independent experiments.

**Fig. 2:**
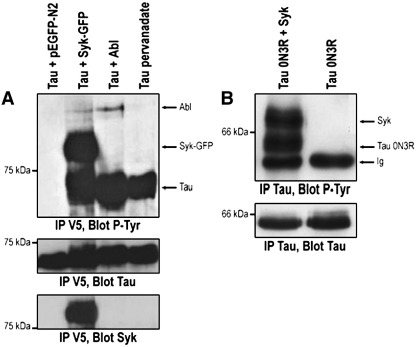
Phosphorylation of tau by Syk in cells. (A) CHO cells were co-transfected with 2N4R V5 tagged human tau and constructs expressing Syk-GFP (Tau + Syk-GFP), Abl (Tau + Abl) or empty vector (Tau + pEGFP-N2) as described previously. An additional positive control consisted of tau-transfected cells treated with pervanadate (Tau pervanadate). Cells were harvested after 24 h and tau was immunoprecipitated using anti-V5 antibody. Western blots on the immunoprecipitates were probed with P-Tyr-100 (IP V5, Blot P-Tyr) and with Tau-5 (IP V5, Blot Tau) or Syk N-19 (IP V5, Blot Syk) after membrane stripping. (B) CHO cells were transiently co-transfected with 0N3R untagged human tau and native human Syk (Tau 0N3R + Syk) or transfected with 0N3R tau alone (Tau 0N3R). Cells were harvested after 24 h and tau was immunoprecipitated using tau-5 antibody. Western blots on the immunoprecipitates were probed with P-Tyr-100 (IP Tau, Blot P-Tyr) and Tau-5 (IP Tau, Blot Tau). The data presented are representative of three independent experiments and were confirmed by using another phosphotyrosine antibody (4G10, Upstate, data not shown).

**Fig. 3:**
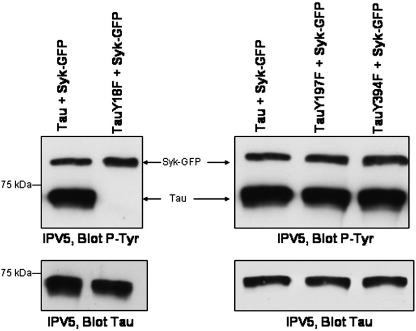
Tyr-18 is the primary site phosphorylated by Syk in transfected CHO cells. CHO cells were transiently co-transfected with Syk-GFP construct and tau (Tau + Syk-GFP) or Y18F mutant tau (Tau Y18F + Syk-GFP) or Y197F mutant tau (Tau Y197F + Syk-GFP) or Y394F mutant tau (Tau Y394F + Syk-GFP), in which tyrosine 18, 197 or 394 were replaced by phenylalanine. The constructs used were based upon tau 2N4R cDNA and were V5-tagged. Cells were harvested after 24 h and tau was immunoprecipitated using anti-V5 antibody. Western blots of the immunoprecipitates were probed with P-Tyr-100 (IPV5, Blot P-Tyr) and Tau-5 (IPV5, Blot Tau). The data presented are representative of three independent experiments and were confirmed by using another phosphotyrosine antibody (4G10, Upstate, data not shown).

**Fig. 4:**
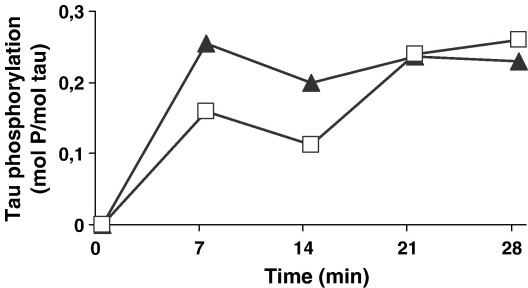
Stoichiometry and time course of phosphate incorporation into tau following phosphorylation with Syk (solid triangles) or Fyn (open squares). Recombinant human tau (2 μg) was incubated with 30 μl of kinase buffer (as in Fig. 1) in the presence of 0.2 M ATP and 5 μCi [γ-32P]ATP. Ten μl aliquots were pipetted in duplicate onto p81 Whatman papers at the indicated times as described previously [22]. The results are representative of two independent experiments.
